# Melatonin prevents bone destruction in mice with retinoic acid–induced osteoporosis

**DOI:** 10.1186/s10020-019-0107-0

**Published:** 2019-08-28

**Authors:** Xudong Wang, Tongzhou Liang, Yuanxin Zhu, Jincheng Qiu, Xianjian Qiu, Chengjie Lian, Bo Gao, Yan Peng, Anjing Liang, Hang Zhou, Xiaoming Yang, Zhiheng Liao, Yongyong Li, Caixia Xu, Peiqiang Su, Dongsheng Huang

**Affiliations:** 1 Department of Orthopedics, Sun Yat-sen Memorial Hospital of Sun Yat-sen University, #107 West Yan Jiang Road, Guangzhou, 510120 Guangdong China; 2 Department of Orthopedics, the First Affiliated Hospital of Sun Yat-sen University, #58 Zhongshan Road II, Guangzhou, 510080 Guangdong China; 3 Research Centre for Translational Medicine, the First Affiliated Hospital of Sun Yat-sen University, Guangzhou, 510080 Guangdong China

**Keywords:** Osteoporosis, Melatonin, Bone metabolism, Bone remodeling, Oxidant stress

## Abstract

**Background:**

The protective effect of melatonin against bone metabolism imbalance in osteoporosis (OP) induced by drugs such as retinoic acid (RA) is unclear. The aim of this study was to explore the role of melatonin in bone destruction based on a mouse model.

**Methods:**

RA-induced OP model mice were established. To assess the effect of melatonin on these mice, micro-CT was used to characterize the trabecular structure of normal mice and those treated with RA (model), RA + low-dose melatonin (Mlt-L), RA + high-dose melatonin (Mlt-H), and RA + alendronate sodium (positive control). The shape of the trabecular bone, the length and diameter of the femoral head and the height and diameter of vertebra(L1) of each group were also measured and the number of osteoclasts was determined by Tartrate-resistant acid phosphatase (TRACP) staining. Meanwhile, the expression of alkaline phosphatase (ALP) was evaluated by immunohistochemistry assays. The differences between groups in terms of liver and kidney oxidation–related indexes and serum and urinary indicators related to bone metabolism were also analyzed. Furthermore, qRT-PCR and western blotting were used to evaluate the effect of melatonin on osteogenic and osteoclastic differentiation in MC3T3-E1 and RAW264.7 cells, respectively.

**Results:**

RA induction led to a decrease in the amount and density of trabecular bone, a decrease in the length and diameter of the femur and height, diameter of the vertebra (L1), a decrease in bone mass and density and the expression of ALP, and an increase in the number of osteoclasts. Melatonin treatment alleviated these effects induced by RA, increasing the amount of trabecular bone in OP mice, improving the microstructure of the femur and vertebra(L1) and increasing bone mass bone density and the expression of ALP, as well as decreasing the number of osteoclasts. Additionally, blood and urinary bone metabolism-related indicators showed that melatonin promoted bone formation and inhibited bone resorption. Determination of oxidant and antioxidant biomarkers in the livers and kidneys of the mice revealed that melatonin promoted the antioxidant level and suppressed the level of oxidant molecules in these organs. In vitro, RA promoted osteoclasts and inhibit osteogenesis by increasing oxidative stress levels in the RAW264.7 and MC3T3-E1 cells, but melatonin reversed this effect. Melatonin may, therefore, play a role in the ERK/SMAD and NF-κB pathways.

**Conclusions:**

Melatonin can alleviate bone loss in RA-induced OP model mice, repair the trabecular microstructure, and promote bone formation. These effects may be related to reducing oxidation levels in vivo and vitro through the ERK/SMAD and NF-κB pathways.

**Electronic supplementary material:**

The online version of this article (10.1186/s10020-019-0107-0) contains supplementary material, which is available to authorized users.

## Background

Osteoporosis (OP) is a systemic disease characterized by low bone mass and micro-architectural deterioration of bone tissue, which increases the risk of bone fracture (Sanchez-Barcelo et al. 2010). It is common in the elderly. Worldwide, approximately 89 million people are diagnosed with OP fracture every year, which is equivalent to one case every 3 s (Johnell and Kanis 2006). OP is divided into two major categories: primary (including mainly postmenopausal and senile OP) and secondary (including mainly endocrine and metabolic OP, disuse osteoporosis, and drug-induced OP). Treatment and care for OP requires enormous human and material resources, resulting in heavy burdens on affected families and society in general. It is believed that the main pathogenesis of OP is an imbalance of bone metabolism, in which osteogenic capacity is decreased and the likelihood of breaking bones is increased (Bonucci and Ballanti 2014). Anti-OP drugs (e.g., bisphosphonate and parathyroid hormone) currently on the market have many disadvantages, such as side effects, high price, poor patient compliance, tumorigenic risks, and an inability to fully restore the balance of bone metabolism.

Melatonin is a neuroendocrine hormone secreted by the pineal gland that has a wide range of effects, including sleep regulation, anti-aging, anti-inflammatory, and anti-oxidation activities (Jarzynka et al. 2009; Zawilska et al. 2009; Witt-Enderby et al. 2006). In the field of orthopedics, it is considered to be a promoter of bone and cartilage development (Zhang et al. 2010a; Gao et al. 2014). Studies have shown that melatonin deficiency leads to an increase in bone resorption, suggesting that it may have an inhibitory effect on osteoclast-related processes (Maria and Witt-Enderby 2014). Ostrowska et al. found that serum melatonin levels were associated with collagen type I, an osteogenesis marker in postmenopausal women, suggesting that decreased melatonin serum levels are associated with increased bone loss in postmenopausal women (Ostrowska et al. 2001a). However, the role of melatonin in OP is not fully understood. A randomized controlled trial suggested that, although there was no significant improvement in bone mineral density (BMD) during a 6-month follow-up period in perimenopausal women treated with melatonin, the melatonin was well tolerated, helped restore the balance of bone remodeling, and prevented loss of bone mass (Kotlarczyk et al. 2012; Maria et al. 2017). Animal studies have also suggested that melatonin helps to regulate bone metabolism. For example, in ovariectomy-induced osteoporosis model mice, melatonin injection reduced urinary deoxypyridinoline (osteogenesis index) and promoted alkaline phosphatase (ALP) activity (osteogenesis index) in blood and bone tissue, confirming its effect in promoting osteogenesis (Ostrowska et al. 2001b).

In the literature, it is noted that melatonin can ameliorate bone metabolism in ovariectomized rats with osteoporosis (Ostrowska et al. 2001b; Uslu et al. 2007). However, recent research has mainly focused on ovariectomy-induced OP models, so there is still a lack of a comprehensive evaluation of the protective effect of melatonin on bone metabolism imbalance in drug-induced OP. In the present study, we constructed a retinoic acid (RA)-induced OP mouse model with intraperitoneal injections of melatonin (low-dose, 5 mg/kg/d; or high-dose, 50 mg/kg/d) or alendronate (10 mg/kg/week) to investigate the effect of melatonin on trabecular microstructure, osteoclast BMD, and oxidation in RA-induced OP mice. Further, the effect of melatonin on osteogenic and osteoclastic differentiation was examined in MC3T3-E1 and RAW264.7 cells, respectively.

## Methods

### Animal model construction and experimental design

A total of 42 female specific-pathogen-free (SPF) C57 mice aged 8 to 9 months were purchased from Charles River Laboratories (Beijing, China) and housed in a metabolic cage under 12 h light/12 h dark conditions. Mice were randomly divided into two groups: control group (*n* = 12) and model group (RA, 70 mg/kg/d, vegetable oil solvent, *n* = 30). After 15 d of administration of RA, six mice in the control group and six in the model group were tested to confirm the success of modeling. Then, mice in the model groups were randomly assigned to four groups (*n* = 6 per group): model group, model + low-melatonin group (5 mg/kg/d, solvent: 5% ethanol in saline), model + high-melatonin group (50 mg/kg/d, solvent: 5% ethanol in saline), and model + alendronate sodium group (10 mg/kg/week, solvent: deionized water), which were then subjected to the corresponding treatment. RA was administered by gavage, and melatonin and alendronate sodium were administered by intraperitoneal injection. The body weight of the mice was recorded weekly for 8 weeks. Two months after modeling, liver and kidney tissues of the mice in each group as well as the left and right tibia and femur were dissected. In addition, blood samples were collected by removing the eyeball. Urine was collected using metabolic cages. The Committee of Institute Research Medical Ethics of Sun Yat-sen University granted approval for this study.

### Measurement of BMD

For the measurement of BMD, mouse femur samples were immersed in PBS at room temperature. X-ray imaging was performed using an X-ray apparatus (SRO-M50; SOFRON, Tokyo, Japan) 15 d after gavage. The BMD was measured by a dual-energy X-ray absorptiometry apparatus (DCS-600R; Aloka, Tokyo, Japan) and expressed as the bone mineral content per unit area.

### Bone microstructure observation

To observe the bone microstructure, mouse femur and vertebra (L1) samples were dissolved in PBS at room temperature. Micro-CT was used for two-dimensional (2D) and three-dimensional (3D) quantitative determination before sacrificing as previously described (Zhang et al. 2010b), and the microstructure parameters of cancellous bone, such as bone volume/total volume (BV/TV), trabecular number (Tb.N), trabecular thickness (Tb.Th), trabecular separation (Tb.Sp), trabecular pattern factor, and cortical bone thickness (Ct.Th), were measured automatically from the Siemens Preclinical Imaging System in a multislice in standard resolution mode (Gong et al. 2005; Hildebrand et al. 1999).

### Organ index, femur length, diameter and vertebra(L1) height, diameter determination

Weights of the liver, spleen, left kidney, right kidney, left tibia, left femur, right tibia, right femur, and the whole body of the mice were determined and used to calculate the organ indexes using the following formula: organ index = mean organ weight (mg)/mean body weight (g) (Yang et al. 2015). In addition, the length diameter of the femur and height, diameter of the vertebra(L1) were measured and recorded.

### Hematoxylin and eosin (HE) staining and immunohistochemical (IHC) staining

Bone samples were fixed with 4% paraformaldehyde, decalcified, dehydrated, embedded in paraffin, and cut into 5 μm slices, after which they were dewaxed by xylene and alcohol (in the following order: 100, 95, 75, and 50%). Slices were immersed in distilled water and HE staining was performed in accordance with standard protocols. Fields with positive staining were captured by microscopy. Additionally, primary antibody anti-ALP (abcam, UK) was incubated overnight at 4 °C and, subsequently, incubated with anti-mouse IgG-HRP (SV-0001; Boster) for 1 h. Then, sections were observed under the microscope (OPTEC CCD TP510) with a 100× objective and pictures were collected. The staining intensity was scored according to previous research (Lian et al. 2019) as follows: 0 (negative), 1 (weakly positive), 2 (moderately positive), and 3 (strongly positive). The percent of positivity was scored according to five categories: 0 (< 5%), 1 (5–25%), 2 (25–50%), 3 (50–75%), and 4 (> 75%). Finally, the percent positivity score was multiplied by the staining intensity score to generate final expression scores, which ranged from 0 to 12.

### Tartrate-resistant acid phosphatase (TRACP) staining

After treatment with melatonin, osteoclasts were fixed in 4% paraformaldehyde and then incubated in TRACP solution including tartaric acid, hexa-azo deoxymethylene red, and naphthol AS-BI phosphate, among others, at 37 °C for 1 h for staining. The staining procedure was carried out in accordance with the manufacturer’s instructions (Jinqiao, Xuzhou, China); TRACP-positive signals were observed and photographed using a microscope. At least four images of 100× magnification were taken at random for each group. The number of TRACP-positive cells in each image was quantified using Image-Pro Plus 6.0 (Media Cybernetics, Inc., MD, USA).

### Oxidation level detection

Liver and kidney tissues were dissected and ground with liquid nitrogen. The resultant supernatant was collected and centrifuged. The levels of malondialdehyde (MDA), superoxide dismutase (SOD), and glutathione peroxidase (GPX) in the tissues of each group were determined using an MDA assay kit (TBA method), a T-SOD assay kit (hydroxylamine method), and a GSH-PX assay kit in accordance with standard instructions (Jiancheng, Nanjing, China).

### Biochemical testing

Blood and urine samples were collected 2 months after establishment of the RA-induced model. Samples were centrifuged at 3000–4000 rpm for 10 min and the supernatant was collected for subsequent analysis. The concentrations of serum calcium, phosphorus, and ALP, as well as urinary phosphorus and urinary calcium, were tested in accordance with kit instructions (Huili, Changchun, China). Serum receptor activator of nuclear factor-κB ligand (RANKL), osteoprotegerin (OPG), and TRACP assays were performed in accordance with the mouse soluble receptor activator of nuclear factor-κB ligand (sRANKL) ELISA kit, mouse osteoprotegerin (OPG) ELISA kit, and mouse TRACP-5b ELISA kit instructions (Cusabio, Changchun, China).

### Cell culture and melatonin application

The RAW264.7 murine monocyte/macrophage cell line and mouse osteoblast cells, MC3T3-E1, were purchased from the Shanghai Cell Bank, Chinese Academy of Sciences (Shanghai, China). Then, 30 ng/ml RANKL (R&D Systems, Minnesota, USA) was added to RAW264.7 cells to initiate differentiation into osteoclast-like cells, which were then cultured with 1 μM RA with or without 100 nM melatonin. To induce osteoblast differentiation, 1 μM RA with or without 100 nM melatonin in α-MEM supplemented with 10% fetal bovine serum (FBS), 50 μg/ml ascorbic acid, and 10 mmol/L β-glycerol phosphate (zqxzbio, Shanghai, China) were added to the MC3T3 cells.

### Quantitative real-time polymerase chain reaction (qRT-PCR)

Total RNA was isolated from cells using Trizol according to the manufacturer’s instructions. The cDNA was synthesized using the PrimeScript RT Master Mix (TaKaRa) according to the manufacturer’s protocols. Real-time qPCR was conducted using a real-time RT-PCR assay (TsingKe, Guangzhou, China) in a final volume of 10 μL according to the manufacturer’s protocol, and the results were analyzed using Applied Biosystems 7500 Fast System SDS software. The expression levels of mRNAs were calculated by the standard 2-△△Ct method with *Gapdh* as internal controls. The primer pairs used are shown in Table 1.

**Table 1:** All primers for reverse transcription-quantitative polymerase chain reaction

Gene	Primer sequence(5′-3′)
*Gapdh*	Forward:AGGTCGGTGTGAACGGATTTG; Reverse: TGTAGACCATGTAGTTGAGGTCA;
*Tnfrsf11a*	Forward: GGACGGTGTTGCAGCAGAT; Reverse: GCAGTCTGAGTTCCAGTGGTA;
*Ctsk*	Forward: GAAGAAGACTCACCAGAAGCAG; Reverse: TCCAGGTTATGGGCAGAGATT;
*Calcr*	Forward:CAAACCGAAGATGAGGTTCCTT; Reverse:TGGGCTCACTAGGAGCAGG;
*Ocn*	Forward: CTGACCTCACAGATCCCAAGC; Reverse:TGGTCTGATAGCTCGTCACAAG;
*Opn*	Forward: ACCCAGAAACTGGTCATCAGC; Reverse:CTGCAATACACACACTCATCACT;
*Runx2*	Forward:ATGCTTCATTCGCCTCACAAA; Reverse:GCACTCACTGACTCGGTTGG;
*Nox1*	Forward: TCTCCAGCCTATCTCATCCTGA; Reverse: GCTGCATACATCACTGTCATGTT;
*Nox2*	Forward: TGTGGTTGGGGCTGAATGTC; Reverse: CTGAGAAAGGAGAGCAGATTTCG;
*Sod1*	Forward:AACCAGTTGTGTTGTCAGGAC; Reverse:CCACCATGTTTCTTAGAGTGAGG;
*Sod2*	Forward: CAGACCTGCCTTACGACTATGG; Reverse: CTCGGTGGCGTTGAGATTGTT;

*Tnfrsf11a* Tumor necrosis factor receptor superfamily member 11a, *Ctsk* Cathepsin K, *Calcr* Calcitonin receptor, *Ocn* Osteocalcin, *Opn* Osteopontin, *Runx2* Runt-related transcription factor-2, *Nox1* NADPH oxidase 1, *Nox2* NADPH oxidase 2, *Sod1* Cu/Zn superoxide dismutase, *Sod2* Manganese superoxide dismutase

### Western blot analysis

The treated cells lysates were prepared using RIPA lysis buffer containing proteinase and phosphatase inhibitors (Sigma-Aldrich, MO, USA). Protein concentration was quantified with a Pierce™ BCA Protein Assay kit (ThermoFisher, MA, USA), and then proteins were separated by 10% SDS-polyacrylamide gel before transfer to a PVDF membrane. The blotting membrane was blocked with TBST containing 5% BSA for 1 h, and then incubated with anti-Rank, anti-Runx2, anti-Sod1, anti-Sod2, anti-p-Erk1/2 (Thr202/Tyr204), anti-Erk1/2, anti-p65, anti-phospho-P65, anti-IκBα, anti-phospho-IκBα, anti-Gapdh (CST, Danvers, MA, USA), anti-Ctsk, anti-Calcr, anti-osteopontin (Opn), anti-osteocalcin (Ocn), anti-Nox1, and anti-Nox2 (Abcam, Cambridge, UK) overnight at 4 °C, followed by incubation with goat anti-rabbit IgG H&L (HRP) or goat anti-mouse IgG H&L (HRP) secondary antibodies (Thermo Scientific, Waltham, MA, USA). Proteins were visualized with an ECL western blot detection system.

### Statistics

The data were analyzed with SPSS 20.0 software (IBM, Armonk, NY, USA) and the results were expressed as the mean ± SD. The *t*-test was used for comparisons between two groups, while one-way analysis of variance was utilized for comparisons among multiple groups, followed by the least significant difference method for post hoc analysis. *P* < 0.05 was considered statistically significant.

## Results

### Construction of OP model induced by RA

**Fig. 1:**
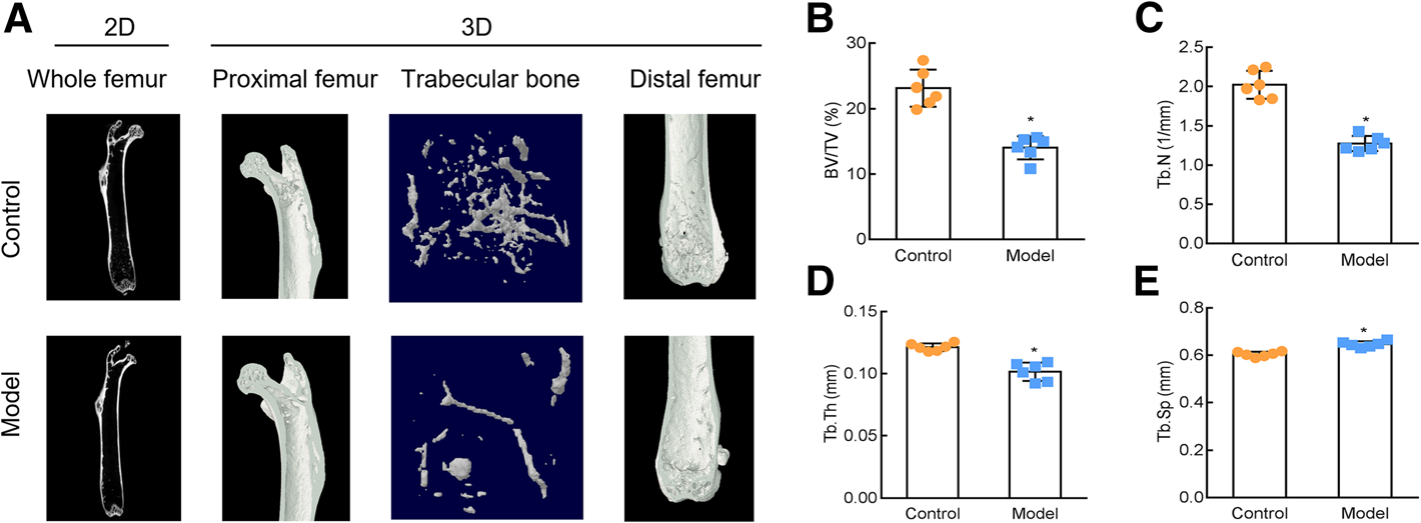
Construction of osteoporosis (OP) model induced by retinoic acid (RA). Mice were subjected to a femoral micro-CT assay of the whole femur (2D) and proximal femur, trabecular bone, and distal femur (3D) 15 d after RA administration (**a**). Bone volume over tissue volume (BV/TV) (**b**), number of trabeculae (Tb.N) (**c**), bone trabecular thickness (Tb.Th) (**d**), and trabecular separation (Tb.Sp) (**e**) were determined 15 d after RA administration. Control: normal mice, Model: RA-induced OP model mice. *, *P* < 0.05 vs control; *n* = 6 per group

The OP model was constructed using RA and subjected to femoral micro-CT testing 15 d after gavage. The 2D and 3D reconstruction micro-CT results showed that the amount of trabecular bone in the model group was reduced and its density was low (Fig. 1a). In addition, BV/TV, Tb. Th, and Tb. N showed significant decrease in the OP model mice, while the value of Tb. Sp was slightly increased (Fig. 1b-e).

**Fig. 2:**
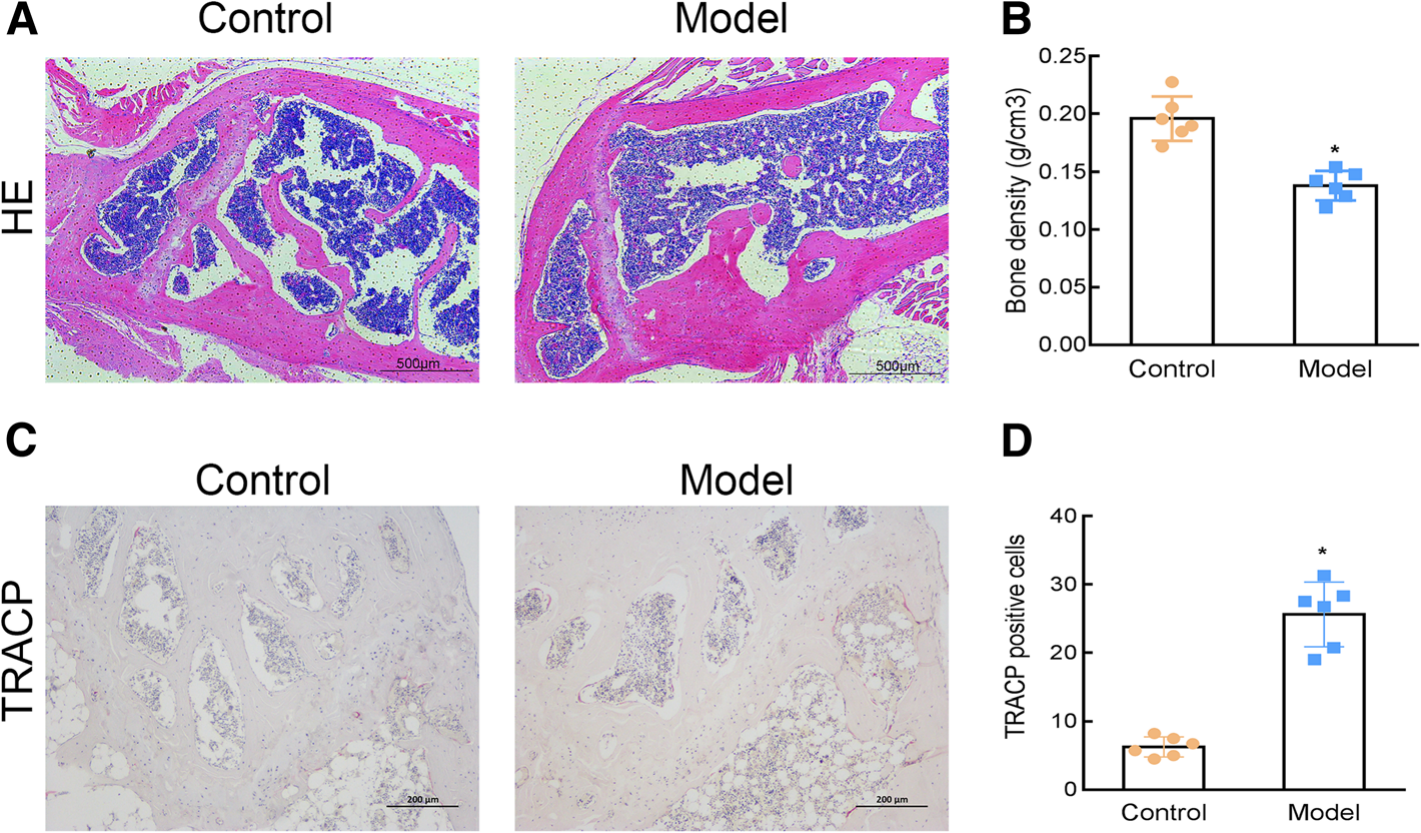
Osteoporosis (OP) was induced in mice with retinoic acid (RA). HE staining (40X) (**a**), femur bone mineral density determination (**b**), and TRACP staining (100X) (**c**) were performed to investigate the changes in femoral trabecular structure, bone density, and number of osteoclasts (**d**) in the OP model. Control: normal mice, Model: RA-induced OP model mice. *, *P* < 0.05 vs control; *n* = 6 per group

HE staining showed that in the model group, the number of femoral trabeculae was reduced and the bone structure had a low density (Fig. 2a). Compared with the normal control group, the bone mass density also significantly decreased in the model group (Fig. 2b). TRACP staining showed a significant increase in the number of TRACP-positive cells in the model group compared with that in the control (Fig. 2c and d), indicating that the OP mouse model had been successfully constructed.

### Melatonin improves femoral and vertebral (L1) changes in OP mice

**Fig. 3:**
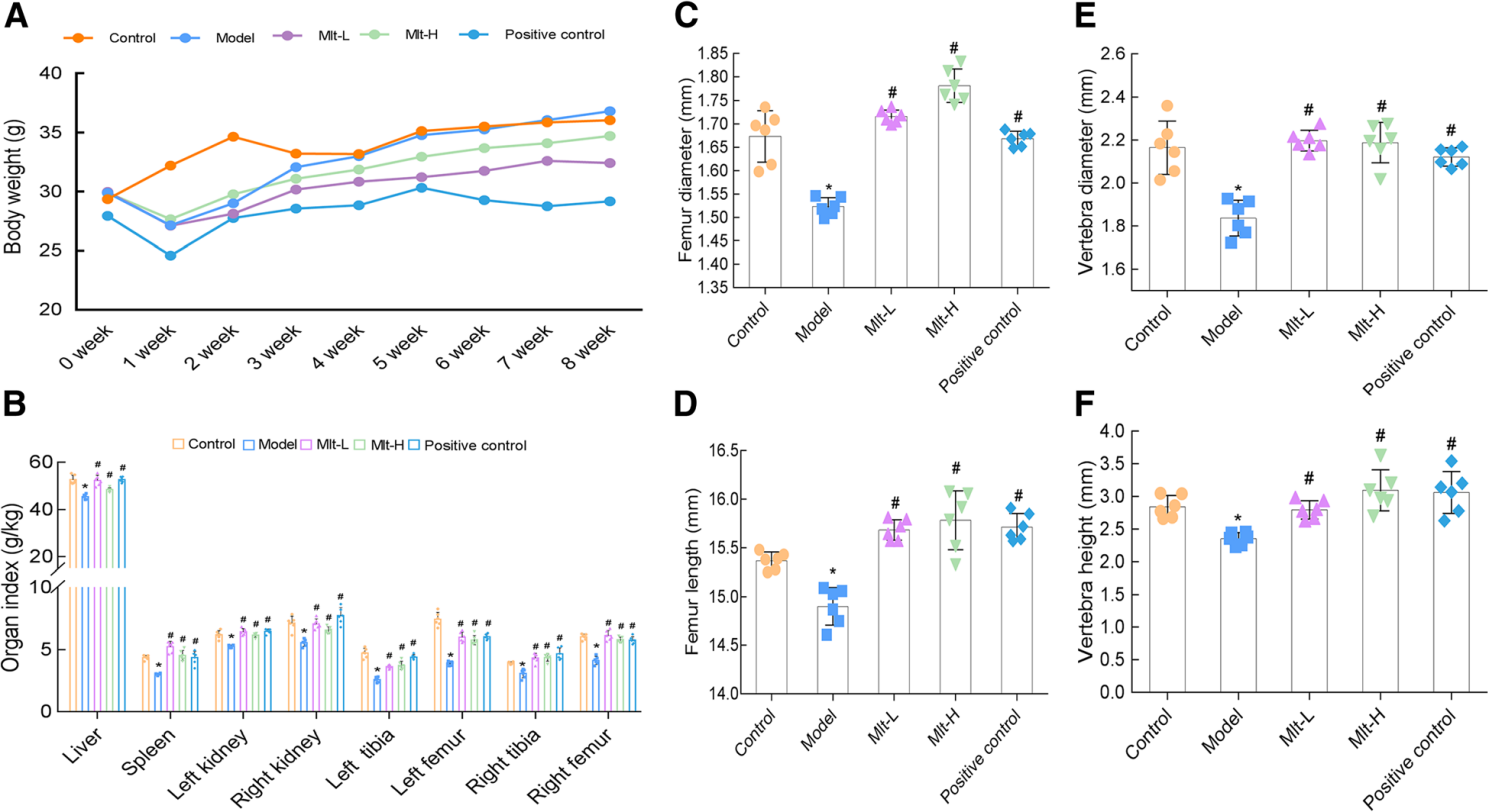
Melatonin improves femoral and vertebral (L1) changes, body weight, and organ index in osteoporosis (OP) mice. Effects of treatment with melatonin at different doses on body weight (**a**), organ index (**b**), femur diameter (**c**), femur length (**d**), vertebra diameter (**e**), and vertebra height (**f**). Control: normal mice, Model: retinoic acid (RA)-induced OP model mice, Mlt-L: low-dose melatonin-treated OP model mice, Mlt-H: high-dose melatonin-treated OP model mice. Positive control: alendronate-treated OP mice. *, *P* < 0.05 vs control. #, *P* < 0.05 vs model; *n* = 6 per group

To study the effect of melatonin on the femurs and vertebra (L1) of OP mice, the control group, a model group, a low-dose melatonin model group (Mlt-L), and a high-dose melatonin model group (Mlt-H) were established, while OP mice treated with alendronate were used as a positive control. During the modeling period, the weight of the mice generally decreased, then gradually increased again after the modeling stopped (Fig. 3a). Analysis of the organ weight index showed that melatonin can reverse changes induced by RA in the weight indexes of the liver, both kidneys, both tibia, and both femurs of mice (Fig. 3b). Measurements showed that the length, diameter of the femur, and height, diameter of the vertebra(L1) were significantly reduced in the model group, and the melatonin treatment (both Mlt-L and Mlt-H) restored the length, diameter of the femur and height, diameter of the vertebra to the same values as the control group (Fig. 3c-f).

**Fig. 4:**
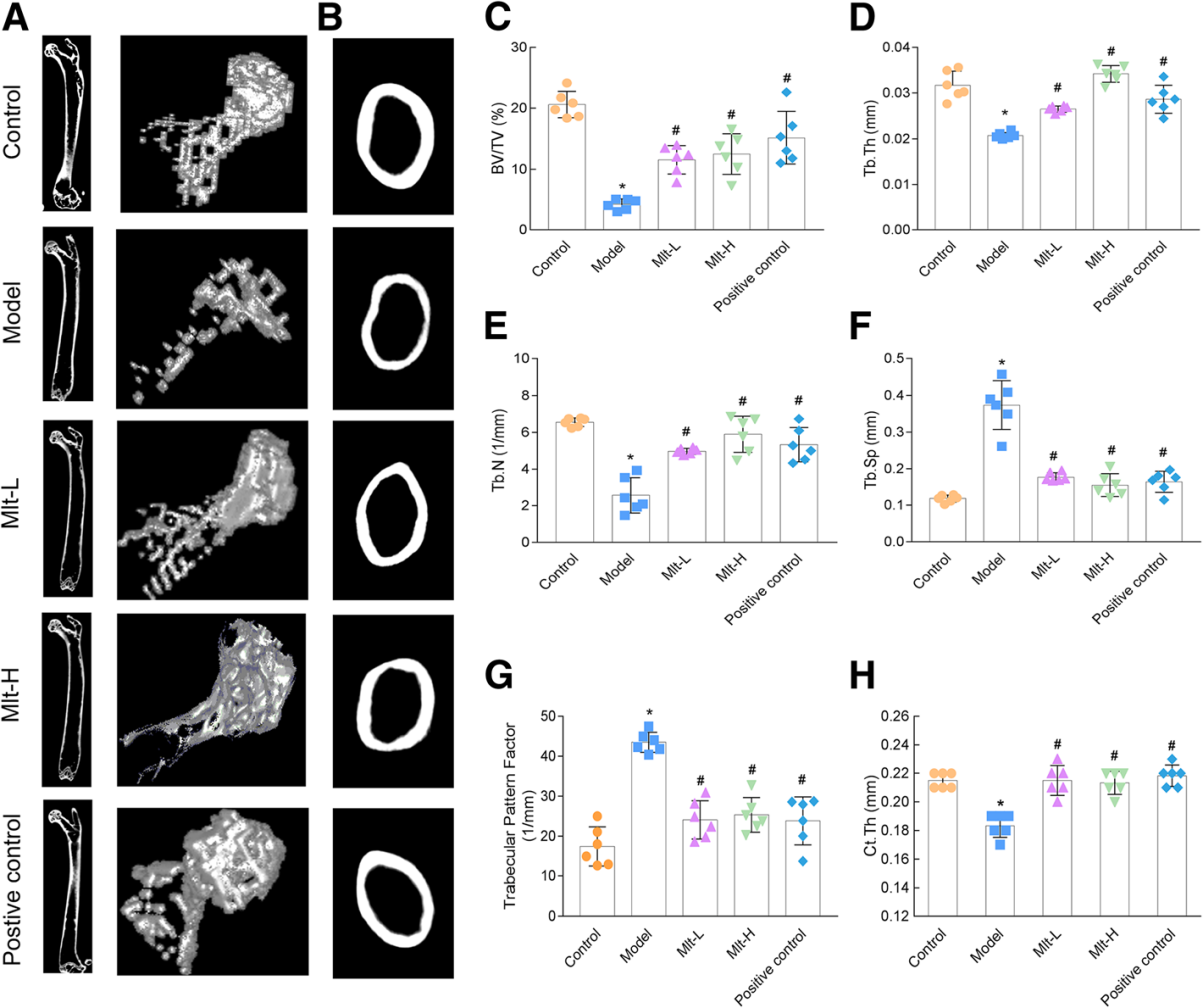
Effect of melatonin on the femur microstructure of osteoporosis (OP) model mice. **a** and **b** Micro-CT detection after the administration of melatonin or alendronate. Images of 2D and 3D reconstruction obtained by micro-CT showed that the model group had a reduction in the number of femoral trabeculae and the thickness of femur diaphysis. Microstructure parameters of BV/TV (**c**), Tb. Th (**d**), Tb. N (**e**), Tb. Sp (**f**), trabecular pattern factor (**g**), and Ct. Th (**h**) were improved in melatonin-treated mice. Control: normal mice, Model: retinoic acid (RA)-induced OP model mice, Mlt-L: low-dose melatonin-treated OP model mice, Mlt-H: high-dose melatonin-treated OP model mice. Positive control: alendronate-treated OP mice. *, *P* < 0.05 vs control. #, *P* < 0.05 vs model; *n* = 6 per group

**Fig. 5:**
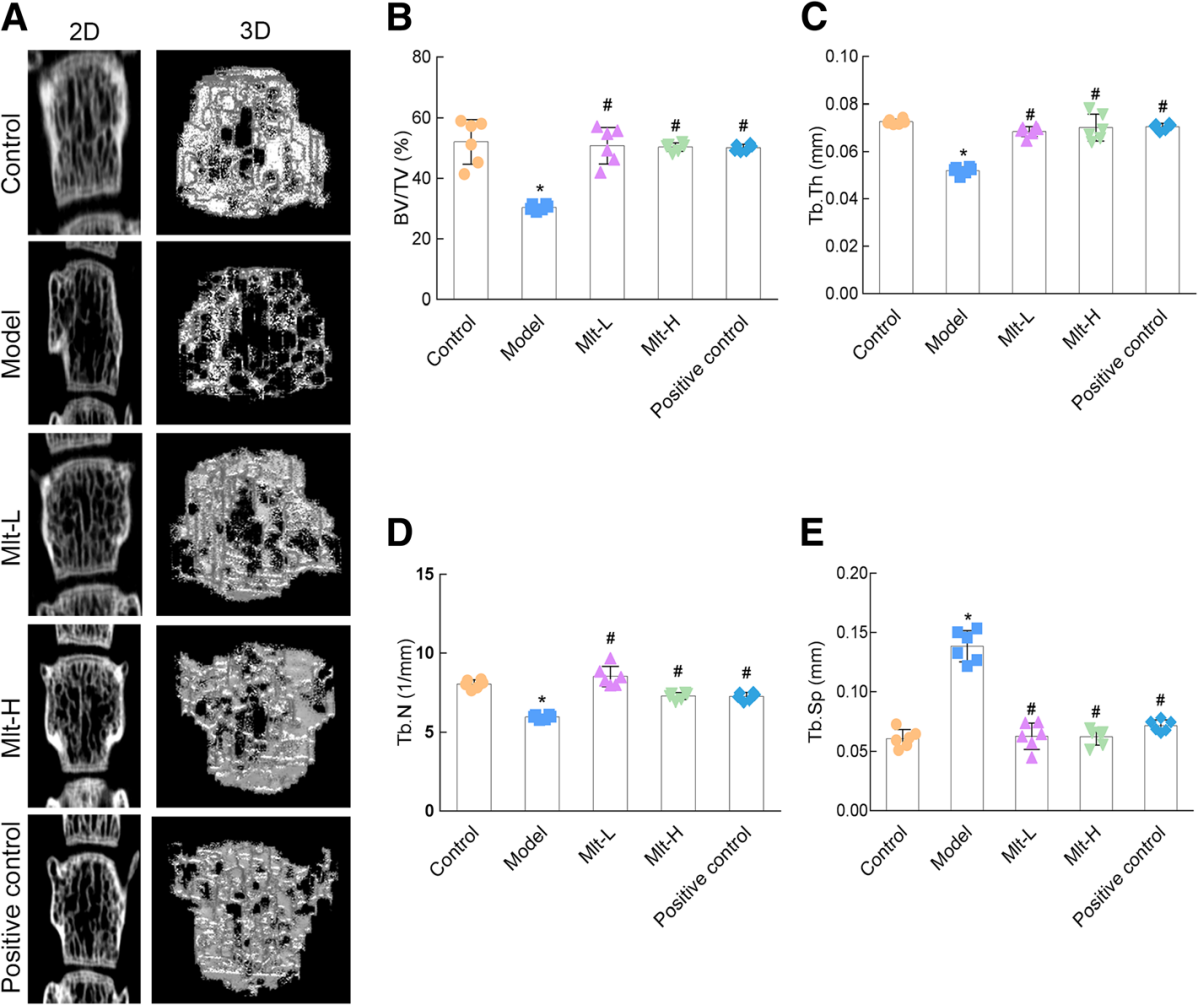
Effect of melatonin on the vertebral (L1) microstructure of osteoporosis (OP) model mice. **a** Micro-CT detection after the administration of melatonin or alendronate. Images of 2D and 3D reconstruction obtained by micro-CT showed that the model group had reduced the number of vertebral trabeculae. Microstructure parameters of BV/TV (**b**), Tb. Th (**c**), Tb. N (**d**), and Tb. Sp (**e**) were improved in melatonin-treated mice. Control: normal mice, Model: retinoic acid (RA)-induced OP model mice, Mlt-L: low-dose melatonin-treated OP model mice, Mlt-H: high-dose melatonin-treated OP model mice. Positive control: alendronate-treated OP mice. *, *P* < 0.05 vs control. #, *P* < 0.05 vs model; *n* = 6 per group

In addition, the trabecular microstructure of the femur and the vertebra (L1) were characterized by micro-CT after the administration of melatonin or alendronate. Micro-CT 2D imaging and 3D reconstructions showed a reduction in the number of femoral and vertebral (L1) trabeculae and the thickness of femur diaphysis in the model group (Figs. 4a, b, and 5a), whereas mice treated with melatonin (both Mlt-L and Mlt-H) had significantly higher BV/TV values than OP model mice (Figs. 4c and 5b). In addition, Tb. Th, Tb. N, and Ct. Th in the Mlt-L and Mlt-H mice were found to be significantly increased (Figs. 4d, e, h and 5c, d) while Tb. Sp and the trabecular pattern factor were significantly decreased (Figs. 4f, g, and 5e) compared to the OP model mice.

### Melatonin improves OP in mice by reducing the number of osteoclasts and increasing the expression of ALP

**Fig. 6:**
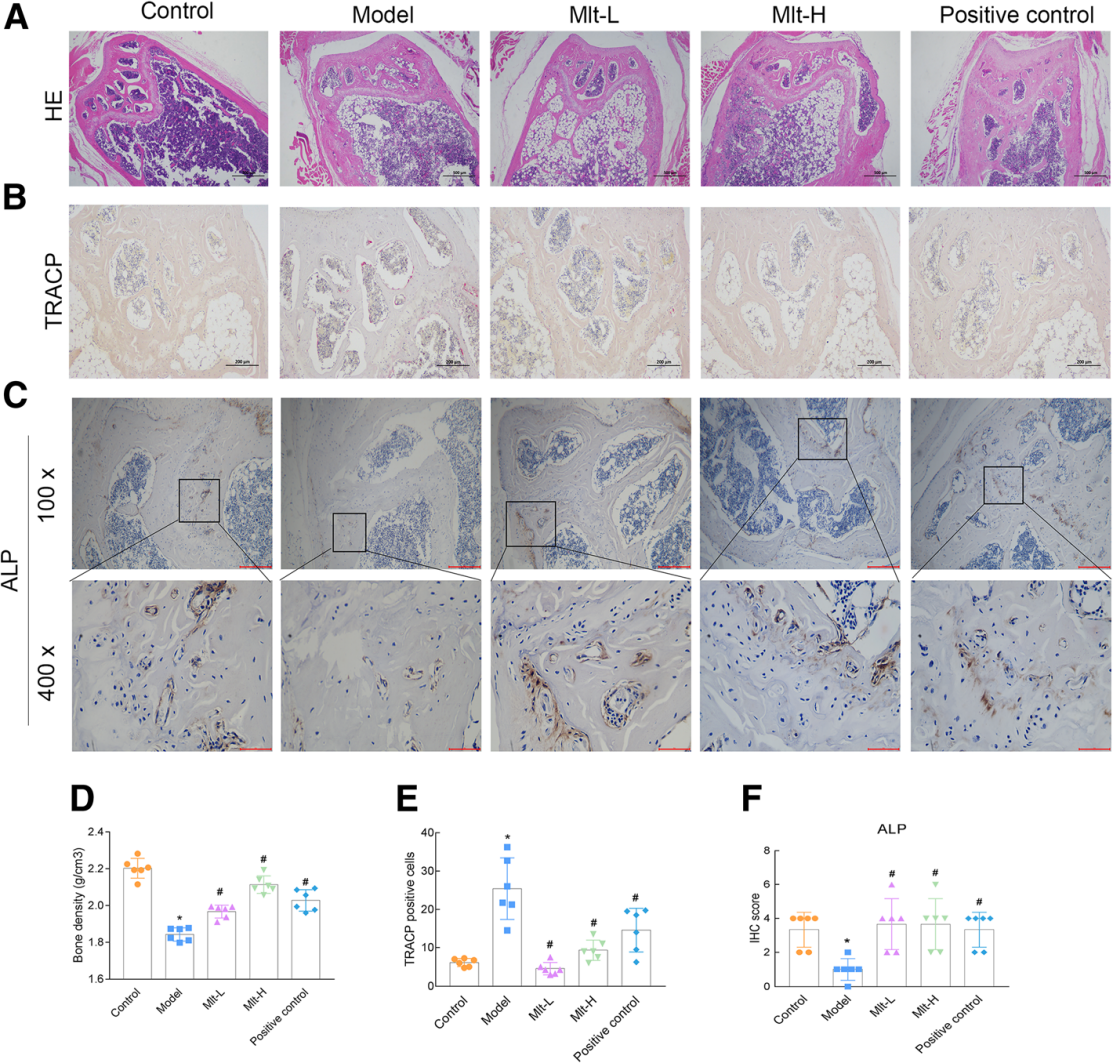
Melatonin improves the femur trabecular bone and reduces the number of osteoclasts of osteoporosis (OP) model mice. After the administration of melatonin or alendronate to trabecular bone for 2 months, distal femur was detected by HE staining (40X) (**a**), TRACP staining (100X) (**b**), and ALP IHC staining (100X and 400X) (**c**). Bone density was assessed (**d**), and the number of TRACP-stained osteoclasts was quantified (**e**). The IHC staining intensity was scored (**f**). Control: normal mice, Model: retinoic acid (RA)-induced OP model mice, Mlt-L: low-dose melatonin-treated OP model mice, Mlt-H: high-dose melatonin-treated OP model mice. Positive control: alendronate-treated OP mice. *, *P* < 0.05 vs control. #, *P* < 0.05 vs model; *n* = 6 per group

To investigate the effect of melatonin on the number of femoral trabecular cells, HE staining, TRACP staining and ALP IHC staining were used to observe pathological changes in the distal femur and trabecular bone. HE staining revealed that the amount of trabecular bone decreased and the bone structure became sparse in OP, and that this condition was improved in the Mlt-L, Mlt-H, and positive control groups, in that the trabecular bone became more abundant (Fig. 6a). TRACP staining showed an increase in the number of osteoclasts in OP mice, which decreased after the administration of melatonin; TRACP-positive signals in the Mlt-L and Mlt-H groups were significantly lower than in the model group and the positive control group, and were similar to that in the control group (Fig. 6b and e). In addition, BMD measurements in each group showed a significant reduction in bone density in OP mice, but melatonin and alendronate treatments restored the bone density of the model mice to the normal control level (Fig. 6d). Further confirming that melatonin could improve OP, the expression of ALP measured by IHC was inhibited in OP mice, but upregulated in the melatonin and alendronate groups (Fig. 6c and f). Additionally, HE staining, TRACP staining, and ALP IHC staining were performed on vertebra, which more comprehensively confirmed that melatonin can improve OP (Additional file 1: Figure S1).

### Melatonin improves bone metabolism and oxidation in OP mice

**Fig. 7:**
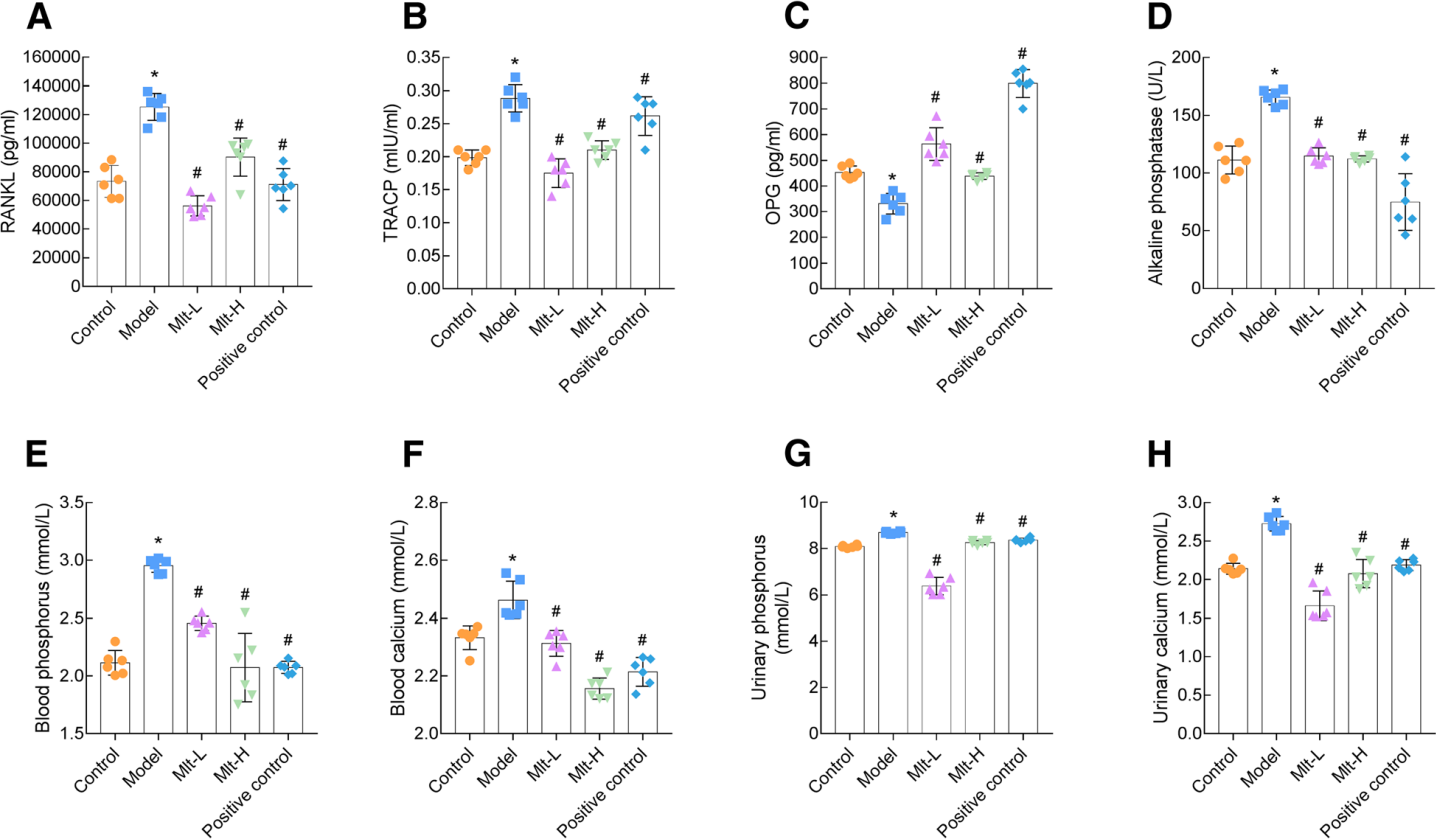
Melatonin improves bone metabolism in osteoporosis (OP) mice. Laboratory tests for biochemical markers of bone metabolism. Serum bone metabolism-related indicators RANKL (**a**), TRACP (**b**), OPG (**c**), ALP (**d**), phosphorus (**e**), calcium (**f**), and urinary phosphorus (**g**) and calcium (**h**) were determined after the administration of melatonin or alendronate for 2 months. Control: normal mice, Model: retinoic acid (RA)-induced OP model mice, Mlt-L: low-dose melatonin-treated OP model mice, Mlt-H: high-dose melatonin-treated OP model mice. Positive control: alendronate-treated OP mice. *, *P* < 0.05 vs control. #, *P* < 0.05 vs model; *n* = 6 per group

After treatment with RA for 15 d, serum and urinary calcium and phosphorus concentration as well as the expression of ALP, RANKL, TRACP and OPG in the serum of mice were tested (Additional file 2: Figure S2). There was no difference in the expression of detection indicators in all mice in the model group. And after randomized treatment with melatonin and alendronate, the corresponding expression was different. The results confirmed that melatonin had a therapeutic effect on RA-induced osteoporosis. In the model group, the concentrations of serum RANKL, TRACP, ALP, phosphorus, and calcium were increased (Fig. 7a, b, and d-f), while OPG (Fig. 7c) was decreased significantly compared to control mice. In both Mlt-L and Mlt-H, these changes were repaired. Urinary sample testing also showed increases in urinary calcium and phosphorus concentrations in the model mice, and melatonin and alendronate treatments reversed this increase in the model group (Fig. 7g and h).

**Fig. 8:**
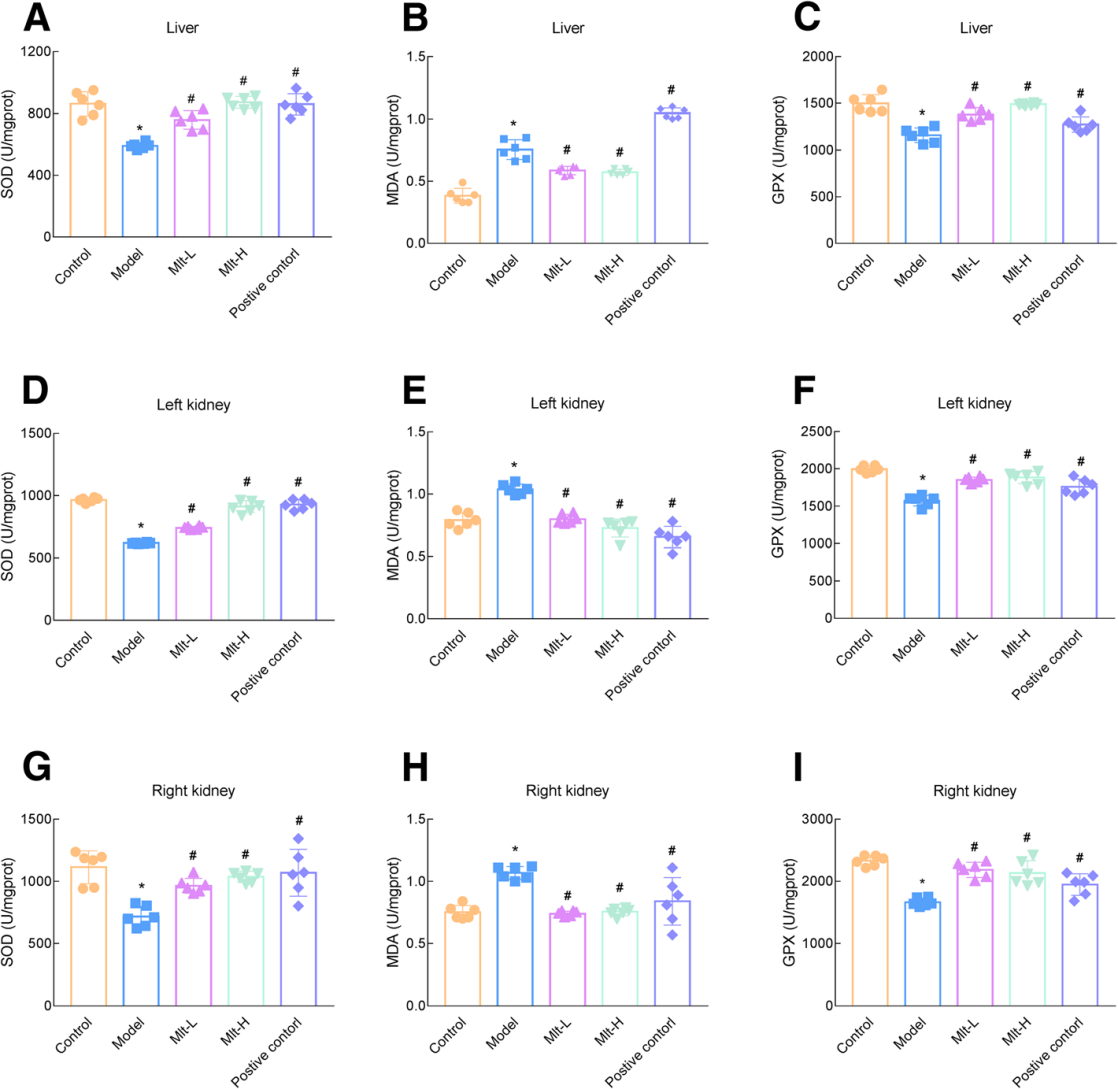
Melatonin improves oxidation in osteoporosis (OP) mice. Oxidative stress-related liver superoxide dismutase (SOD) (**a**), malondialdehyde (MDA) (**b**), and glutathione peroxidase (GPX) (**c**); left kidney SOD (**d**), MDA (**e**), and GPX (**f**); and right kidney SOD (**g**), MDA (**h**), and GPX (**i**) were determined after the administration of melatonin or alendronate for 2 months. Control: normal mice, Model: retinoic acid (RA)-induced OP model mice, Mlt-L: low-dose melatonin-treated OP model mice, Mlt-H: high-dose melatonin-treated OP model mice. Positive control: alendronate-treated OP mice. *, *P* < 0.05 vs control. #, *P* < 0.05 vs model; *n* = 6 per group

In addition, the oxidation level of mouse livers and kidneys was measured. The level of MDA was increased and the levels of GPX and SOD were significantly decreased in model mice. Meanwhile, in both Mlt-L and Mlt-H as well as alendronate-treated mice, the level of MDA was suppressed and the levels of GPX and SOD were restored to those of the control group (Fig. 8).

### Melatonin can inhibit osteoclast differentiation and promote osteogenic differentiation in vitro

**Fig. 9:**
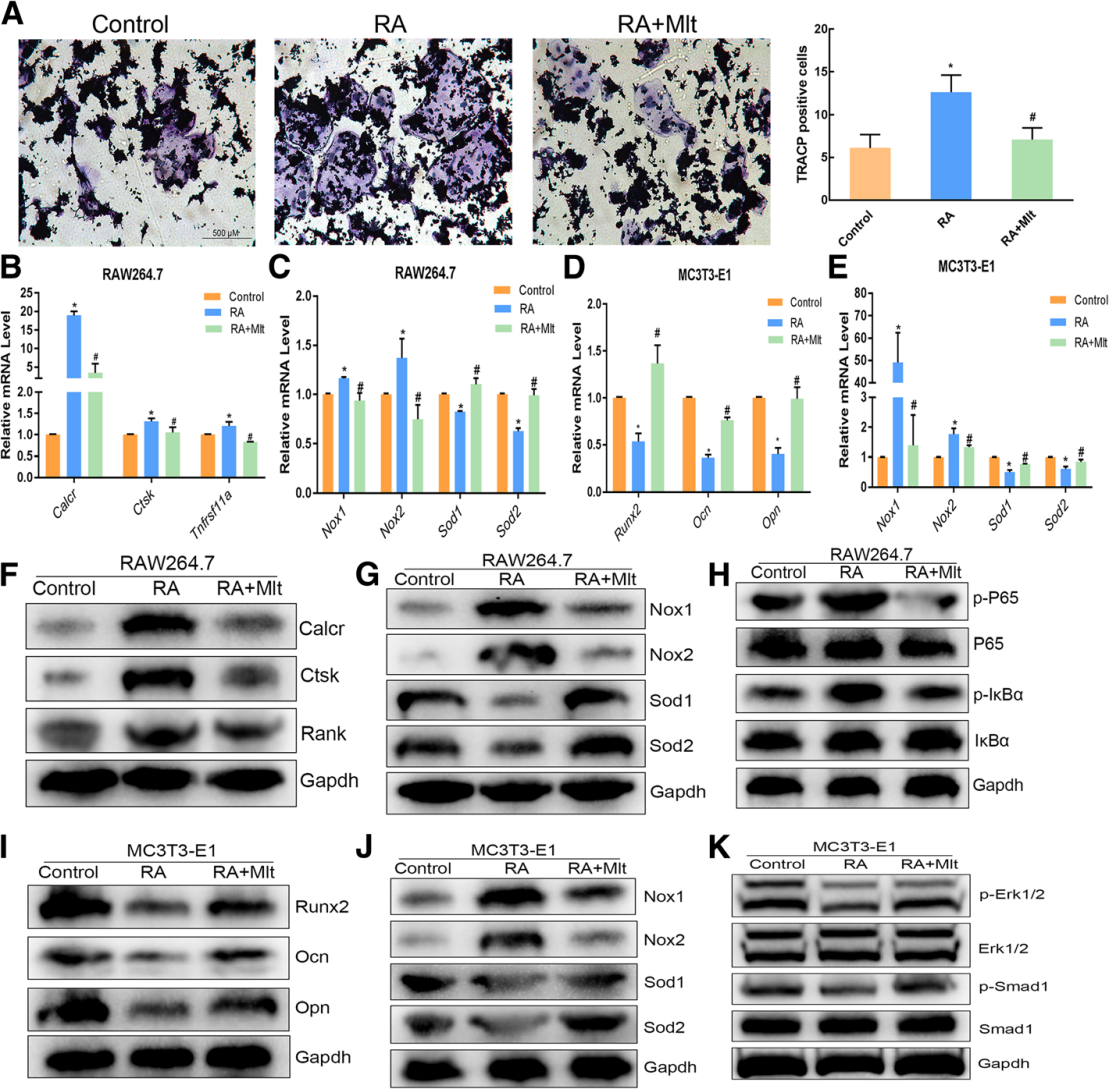
Melatonin can inhibit osteoclast differentiation and promote osteogenic differentiation in vitro. RANKL-induced RAW264.7 cells were cultured with 1 μM retinoic acid (RA) in the presence or absence of 100 nM melatonin. MC3T3-E1 cells were cultured with osteogenic medium with or without RA in the presence or absence of 100 nM melatonin. RAW264.7 cells were stained with TRACP and the number of TRACP-stained osteoclasts was quantified (**a**). The expression levels of Calcr, Ctsk, Rank, Nox1, Nox2, Sod1, and Sod2 were examined by RT-PCR and western blot (**b**, **c**, **f** and **g**). Then, the expression levels of Runx2, Ocn, Opn, Nox1, Nox2, Sod1 and Sod2 in MC3T3-E1 cells were examined by qRT-PCR and Western blot (**d**, **e**, **i** and **j**). Signaling pathway related proteins (p-p65/p65, p-IκBα/IκBα, p-Erk/Erk, and p-Smad1/Smad1) were examined by western blot (**h** and **k**). Control: RANKL-induced RAW264.7 and osteogenic medium induced MC3T3-E1 cells; RA: RA-induced cells; RA + Mlt: melatonin-treated cells. *, *P* < 0.05 vs control. #, *P* < 0.05 vs RA

To investigate the effect of melatonin on osteogenic and osteoclastic differentiation in vitro in RAW264.7 and MC3T3-E1 cells, respectively, were induced. TRACP staining showed an increase in the number of osteoclasts in the RA-induced RAW264.7 cells with RANKL, but the number of osteoclasts was decreased in the RA + Mlt groups (Fig. 9a). Further, markers of osteoclast differentiation (Calcr, Ctsk, and Rank) and oxidation indexes (Nox1 and Nox2) were upregulated, as detected by qPCR and western blot, and antioxidant enzymes (Sod1 and Sod2) were downregulated in RA-induced RAW264.7 cells. However, melatonin could reverse these changes (Fig. 9b, c, f, and g).

Meanwhile, we induced osteogenic differentiation in MC3T3-E1 cells and studied the effect of melatonin on osteogenic differentiation. QPCR and western blot results showed that melatonin could increase the markers of osteogenic differentiation (Runx2, Opn, and Ocn) and antioxidant enzymes (Sod1 and Sod2) and inhibit the expression of oxidation enzymes (Nox1 and Nox2) in RA-induced MC3T3-E1 cells (Fig. 9d, e, i, and j). Finally, we studied the signaling pathways involved and found that RA could upregulate the expression of p-p65 and p-IκBα in RAW264.7 induced by RANKL, whereas melatonin downregulated this expression (Fig. 9h). When osteogenic differentiation was induced in MC3T3-E1 cells with RA, the expression of p-ERK and p-SMAD1 were decreased, but melatonin reversed this (Fig. 9k).

## Discussion

Osteoporosis is a systemic bone disease that results in thinned bone mass, altered microstructure of bone tissues, and higher risk of fracture. RA and ovariectomy (OVX) treatments are frequently used to induce osteoporosis. Compared with the long and complicated experimental process of the OVX model, the RA-induced osteoporosis model is simple, quick, and highly successful, and shows the typical symptoms of osteoporosis (Cowan et al. 2005; Wei et al. 2007; Oršolić et al. 2018). As a derivative of vitamin A and an anti-tumor drug, RA can activate osteoclasts and promote bone absorption (Yang et al. 2016). Meanwhile, it has been reported that RA can damage the ovarian function, decrease the estrogen level, and also affect the occurrence of osteoporosis (Zhao et al. 2016).

In recent years, the RA-induced OP model has been widely used in drug-induced secondary OP studies (Yang et al. 2016), especially in the research and development of Chinese traditional medicine. Using RA-induced animal models, previous studies found that nylestriol/levonorgestrel (Liao et al. 2003), naringin, and quercetin play beneficial roles in protecting against RA-induced OP in rats (Wei et al. 2007; Oršolić et al. 2018). In addition, deer tendon collagen and isoflavone-enriched soy protein have also been found to exert protective effects in an RA-induced mouse model (Zhang et al. 2010c; Yang et al. 2016).

Accumulating evidence shows that melatonin has protective effects against OP in menopausal women. However, as previous studies mainly focused on the role of melatonin in diabetes-induced OP and ovariectomy-induced OP, its role in drug-induced OP models remained unclear. In the current study, by constructing an RA-induced OP mouse model developed to investigate drug-induced OP (Yang et al. 2016), we analyzed differences in trabecular bone, bone metabolism markers, and liver and kidney oxidative stress between groups of mice (normal mice, RA-induced model group, low-dose, and high-dose melatonin-treated groups, and a sodium alendronate-treated group as a positive control) and found that RA induction can lead to changes in trabecular morphology and microstructure, decreased bone density and the expression of ALP, and increased osteoclasts. Meanwhile, melatonin administration can alleviate the effects of RA, rebuilding trabecular bone structure, increasing bone mass, bone density and the expression of ALP, and decreasing the number of osteoclasts. Our results suggest that melatonin has a treatment effect against drug-induced OP. Additionally, our study established a positive control in which mice were treated with alendronate, a drug thought to protect against bone loss and reduce the incidence of fractures in menopausal women (Saag et al. 2017; Liberman et al. 1995). According to a number of indicators, melatonin worked similarly to alendronate. This suggests the therapeutic function and clinical value of melatonin in drug-induced OP bone metabolism imbalance.

Micro-architectural deterioration of bone tissues and low bone mass are two characteristics of OP. Another study has shown that the RA-induced OP model exhibits bone loss and bone structural changes within 1–3 weeks (Fahmy and Soliman 2009). Melatonin treatment of ovariectomized model OP mice has been reported to improve bone microstructure (Uslu et al. 2007). Our current IHC assay and micro-CT data provide evidence that bone microstructure and bone density changed after 15 d of RA induction; moreover, we found that bone structure and bone metabolism imbalance were improved after melatonin treatment. These observations suggest that melatonin has beneficial effects on bone microstructure and enhances bone density, which is consistent with the research results of Sharan et al. (2017). Their results revealed that oral administration of melatonin can increase bone mass and reverse the OVX-induced microstructural and functional degeneration of bone through increasing bone formation.

Various types of cells, particularly osteoclasts and osteoblasts, are involved in the bone remodeling process. Factors such as RANKL and its decoy receptor, OPG, which is secreted from osteoblasts (Martin and Sims 2015), are essential for promoting osteoclast differentiation and inhibiting bone formation. OP is one of the results of an imbalance in bone remodeling. As reviewed by Sanchez-Barcelo et al. (2010), the mechanism of action of melatonin in protecting against OP mainly involves the following aspects: enhanced osteoblast differentiation and activity; suppressed differentiation of osteoclasts; and the clearing of free radicals produced by osteoclasts and responsible for bone resorption. When bone resorption occurs more rapidly than bone formation, bone loss occurs (Isomura et al. 2004). Bone resorptive markers secreted by osteoclasts such as TRACP-5b (Kuo and Chen 2017; Liu et al. 2015), a commonly used biomarker reflecting bone formation, and ALP are upregulated in OP patients and RA-induced animal models (Oršolić et al. 2018; Fink et al. 2016). Consistent with previous studies, the current study revealed that the expression levels of RANKL, which promotes osteoclast differentiation; TRACP-5b, which promotes osteoclast-mediated bone resorption; and ALP, which inhibits osteoclast differentiation, were upregulated, and that these changes were reversed in OP mice treated with melatonin. These findings indicate that melatonin inhibits bone resorption and osteoclastic differentiation in RA-induced OP mice. Additionally, TRACP staining showed that melatonin reduced the number of osteoclasts in the femurs of OP mice. Meanwhile, the effect of melatonin on the osteoclast differentiation of RAW264.7 cells and osteogenic differentiation of MC3T3-E1 cells were performed. We found that melatonin could inhibit the expression of the markers of osteoclast differentiation (Calcr, Ctsk, and Rank) in the RA-induced RAW264.7 cells, but increase the markers of osteogenic differentiation (Runx2, Opn, and Ocn) in the RA-induced MC3T3-E1 cells. Previous study has showed that melatonin could promote human adult mesenchymal stem cells (hAMSCs) differentiation into osteoblasts through an increase in mRNA expression of osteogenic genes Runx2, Bmp-2, and Osteocalcin and this may promote bone formation (Sethi et al. 2010). Therefore, it is possible that melatonin promotes bone remodeling in OP mice by inhibiting osteoclast differentiation and promoting osteogenic differentiation.

Calcium and phosphorus are important bone minerals. Evidence has shown that patients with OP have a high incidence of hypercalciuria (El-Husseini et al. 2017). RA-induced OP animal models also exhibited elevated levels of serum calcium and phosphorus in the present study, which may be related to the increase in the number of osteoclasts in OP; the increase in osteoclasts promotes bone resorption and bone mineral dissolution, decreases bone calcium and phosphorus levels, and increases serum and urinary levels of calcium and phosphorus. Increased urinary calcium excretion suggests increased bone resorption. The application of melatonin reduces the levels of serum and urinary calcium and phosphorus, indicating that melatonin alleviates OP.

Oxidative stress plays an important role in the formation of bone. Excessive free radical production and aggregation can induce oxidative stress, cause lipid peroxidation, inhibit osteogenic differentiation, and promote bone resorption (Bai et al. 2004; Wauquier et al. 2009; Callaway and Jiang 2015). MDA, the end-product of important oxygen free lipid peroxidation, is an important marker of the degree of damage of reactive oxygen free radicals (Spirlandeli et al. 2014). Studies have found that it is significantly elevated in the serum of women with OP after menopause (Ozgocmen et al. 2007; Sendur et al. 2009). The antioxidant enzymes SOD and GPX scavenge free radicals, and their levels have been found to be significantly lower in the serum of women with OP after menopause than in healthy postmenopausal women (Sharma et al. 2015). If excessive free radical production exceeds the coping ability of the natural antioxidant defense mechanism, bone resorption may occur (Sheweita and Khoshhal 2007). In the present study, we found that MDA increased in the model group, suggesting an increase in oxidative stress and decreases in SOD and GPX, in turn suggesting a decrease in antioxidant levels. In contrast, SOD and GPX in the melatonin-treated group were significantly improved, suggesting that melatonin has an anti-oxidative effect, possibly due to scavenging free radicals, in the RA-induced OP model (Orsolic et al. 2014).

## Conclusions

In summary, melatonin can improve the RA-induced trabecular microstructure of OP model mice, inhibit osteoclast differentiation, and promote bone formation. These effects may be related to the reduction of oxidative stress in vivo and vitro through the ERK/SMAD and NF-κB pathways.

## Additional files

Additional file 1: **Figure S1.** Melatonin improves the vertebral (L1) trabecular bone and reduces the number of osteoclasts of osteoporosis (OP) model mice. After the administration of melatonin or alendronate to trabecular bone for 2 months, vertebra were examined by HE staining (40X) (**A**), TRACP staining (100X) (**B**), and ALP IHC staining (100X and 400X) (**C**). The number of TRACP-stained osteoclasts was quantified (**D**) and the IHC staining intensity was scored (**E**). Control: normal mice, Model: retinoic acid (RA)-induced OP model mice, Mlt-L: low-dose melatonin-treated OP model mice, Mlt-H: high-dose melatonin-treated OP model mice. Positive control: alendronate-treated OP mice. *, *P* < 0.05 vs control. #, *P* < 0.05 vs model; *n* = 6 per group. (TIF 8735 kb)

Additional file 2: **Figure S2.** Biochemical markers bone metabolism in osteoporosis (OP) mice. Laboratory tests for biochemical markers of bone metabolism. Serum bone metabolism-related indicators RANKL (**A**), TRACP (**B**), OPG (**C**), ALP (**D**), phosphorus (**E**), calcium (**F**), and urinary phosphorus (**G**), and calcium (**H**) were determined after the administration of retinoic acid (RA) for 15 d. Control: normal mice, Model: RA-induced OP model mice, Mlt-L: low-dose melatonin-treated OP model mice, Mlt-H: high-dose melatonin-treated OP model mice. Positive control: alendronate-treated OP mice. *, *P* < 0.05 vs control. #, *P* < 0.05 vs model; *n* = 6 per group. (TIF 1247 kb)

## Data Availability

All relevant data supporting the conclusions of this article is included within the manuscript.

## Abbreviations

ALP: Alkaline phosphatase
BV/TV: Bone volume/Total volume
Calcr: Calcitonin receptor
Ct.Th: Cortical bone thickness
Ctsk: Cathepsin K
GPX: Glutathione peroxidase
HE: Hematoxylin and eosin
MDA: Malondialdehyde
Mlt-H: High-dose melatonin
Mlt-L: Low-dose melatonin
Nox1: NADPH oxidase 1
Nox2: NADPH oxidase 2
Ocn: Osteocalcin
OP: Osteoporosis
OPG: Osteoprotegerin
Opn: Osteopontin
RA: Retinoic acid
RANKL: Receptor activator of nuclear factor-κB ligand
Runx2: Runt-related transcription factor-2
SOD: Superoxide dismutase
Sod1: Cu/Zn superoxide dismutase
Sod2: Manganese superoxide dismutase
SPF: Specific-pathogen-free
sRANKL: Soluble receptor activator of nuclear factor-κB ligand
Tb.N: Trabecular number
Tb.Sp: Trabecular separation
Tb.Th: Trabecular thickness
Tnfrsf11a: Tumor necrosis factor receptor superfamily member 11a
TRACP: Tartrate-resistant acid phosphatase
TRACP-5b: Tartrate-resistant acid phosphatase-5b

## References

[CR1] Bai XC, Lu D, Bai J, Zheng H, Ke ZY, Li XM (2004). Oxidative stress inhibits osteoblastic differentiation of bone cells by ERK and NF-kappaB. Biochem Biophys Res Commun.

[CR2] Bonucci E, Ballanti P (2014). Osteoporosis-bone remodeling and animal models. Toxicol Pathol.

[CR3] Callaway DA, Jiang JX (2015). Reactive oxygen species and oxidative stress in osteoclastogenesis, skeletal aging and bone diseases. J Bone Miner Metab.

[CR4] Cowan CM, Aalami OO, Shi Y, Chou Y, Mari C, Thomas R (2005). Bone morphogenetic protein 2 and retinoic acid accelerate in vivo bone formation, osteoclast recruitment, and bone turnover. Tissue Eng.

[CR5] El-Husseini A, Chakraborty A, Yuan Q, Inayatullah S, Bush H, Sawaya BP (2017). Urinary calcium excretion and bone turnover in osteoporotic patients. Clin Nephrol.

[CR6] Fahmy SR, Soliman AM (2009). Oxidative stress as a risk factor of osteoporotic model induced by vitamin A in rats. Aust J Basic Appl Sci.

[CR7] Fink HA, Litwack-Harrison S, Taylor BC, Bauer DC, Orwoll ES, Lee CG (2016). Clinical utility of routine laboratory testing to identify possible secondary causes in older men with osteoporosis: the osteoporotic fractures in men (MrOS) study. Osteoporos Int.

[CR8] Gao W, Lin M, Liang A, Zhang L, Chen C, Liang G (2014). Melatonin enhances chondrogenic differentiation of human mesenchymal stem cells. J Pineal Res.

[CR9] Gong H, Zhang M, Yeung HY, Qin L (2005). Regional variations in microstructural properties of vertebral trabeculae with aging. J Bone Miner Metab.

[CR10] Hildebrand T, Laib A, Müller R, Dequeker J, Rüegsegger P (1999). Direct 3-D morphometric analysis of human cancellous bone: microstructural data from spine, femur, iliac crest and calcaneus. J Bone Miner Res.

[CR11] Isomura H, Fujie K, Shibata K, Inoue N, Iizuka T, Takebe G (2004). Bone metabolism and oxidative stress in postmenopausal rats with iron overload. Toxicology.

[CR12] Jarzynka MJ, Passey DK, Johnson DA, Konduru NV, Fitz NF, Radio NM (2009). Microtubules modulate melatonin receptors involved in phase-shifting circadian activity rhythms: in vitro and in vivo evidence. J Pineal Res.

[CR13] Johnell O, Kanis JA (2006). An estimate of the worldwide prevalence and disability associated with osteoporotic fractures. Osteoporos Int.

[CR14] Kotlarczyk MP, Lassila HC, O'Neil CK, D'Amico F, Enderby LT, Witt-Enderby PA (2012). Melatonin osteoporosis prevention study (MOPS): a randomized, double-blind, placebo-controlled study examining the effects of melatonin on bone health and quality of life in perimenopausal women. J Pineal Res.

[CR15] Kuo TR, Chen CH (2017). Bone biomarker for the clinical assessment of osteoporosis: recent developments and future perspectives. Biomark Res.

[CR16] Lian C, Wang X, Qiu X (2019). Collagen type II suppresses articular chondrocyte hypertrophy and osteoarthritis progression by promoting integrin β1− SMAD1 interaction. Bone Res.

[CR17] Liao EY, Luo XH, Wang WB, Wu XP, Zhou HD, Dai RC (2003). Effects of different nylestriol/levonorgestrel dosages on bone metabolism in female Sprague-Dawley rats with retinoic acid-induced osteoporosis. Endocr Res.

[CR18] Liberman UA, Weiss SR, Broll J, Minne HW, Quan H, Bell NH (1995). Effect of oral alendronate on bone mineral density and the incidence of fractures in postmenopausal osteoporosis. The alendronate phase III osteoporosis treatment study group. N Engl J Med.

[CR19] Liu RH, Kang X, Xu LP, Nian HL, Yang XW, Shi HT (2015). Effects of the combined extracts of Herba Epimedii and Fructus Ligustri Lucidi on bone mineral content and bone turnover in osteoporotic rats. BMC Complement Altern Med.

[CR20] Maria S, Swanson MH, Enderby LT, D'Amico F, Enderby B, Samsonraj RM (2017). Melatonin-micronutrients osteopenia treatment study (MOTS): a translational study assessing melatonin, strontium (citrate), vitamin D3 and vitamin K2 (MK7) on bone density, bone marker turnover and health related quality of life in postmenopausal osteopenic women following a one-year double-blind RCT and on osteoblast-osteoclast co-cultures. Aging (Albany NY).

[CR21] Maria S, Witt-Enderby PA (2014). Melatonin effects on bone: potential use for the prevention and treatment for osteopenia, osteoporosis, and periodontal disease and for use in bone-grafting procedures. J Pineal Res.

[CR22] Martin TJ, Sims NA (2015). RANKL/OPG; critical role in bone physiology. Rev Endocr Metab Disord.

[CR23] Orsolic N, Goluza E, Dikic D, Lisicic D, Sasilo K, Rodak E (2014). Role of flavonoids on oxidative stress and mineral contents in the retinoic acid-induced bone loss model of rat. Eur J Nutr.

[CR24] Oršolić N, Jeleč Ž, Nemrava J, Balta V, Gregorović G, Jeleč D (2018). Effect of quercetin on bone mineral status and markers of bone turnover in retinoic acid-induced osteoporosis. Pol J Food Nutr Sci.

[CR25] Ostrowska Z, Kos-Kudla B, Marek B, Swietochowska E, Gorski J (2001). Assessment of the relationship between circadian variations of salivary melatonin levels and type I collagen metabolism in postmenopausal obese women. Neuro Endocrinol Lett.

[CR26] Ostrowska Z, Kos-Kudla B, Swietochowska E, Marek B, Kajdaniuk D, Gorski J (2001). Assessment of the relationship between dynamic pattern of nighttime levels of melatonin and chosen biochemical markers of bone metabolism in a rat model of postmenopausal osteoporosis. Neuro Endocrinol Lett.

[CR27] Ozgocmen S, Kaya H, Fadillioglu E, Aydogan R, Yilmaz Z (2007). Role of antioxidant systems, lipid peroxidation, and nitric oxide in postmenopausal osteoporosis. Mol Cell Biochem.

[CR28] Saag KG, Petersen J, Brandi ML, Karaplis AC, Lorentzon M, Thomas T (2017). Romosozumab or alendronate for fracture prevention in women with osteoporosis. N Engl J Med.

[CR29] Sanchez-Barcelo EJ, Mediavilla MD, Tan DX, Reiter RJ (2010). Scientific basis for the potential use of melatonin in bone diseases: osteoporosis and adolescent idiopathic scoliosis. J Osteoporos.

[CR30] Sendur OF, Turan Y, Tastaban E, Serter M (2009). Antioxidant status in patients with osteoporosis: a controlled study. Joint Bone Spine.

[CR31] Sethi S, Radio NM, Kotlarczyk MP, Chen CT, Wei YH, Jockers R, Witt-Enderby PA (2010). Determination of the minimal melatonin exposure required to induce osteoblast differentiation from human mesenchymal stem cells and these effects on downstream signaling pathways. J Pineal Res.

[CR32] Sharan K, Lewis K, Furukawa T, Yadav V (2017). Regulation of bone mass through pineal-derived melatonin-MT 2 receptor pathway. J Pineal Res.

[CR33] Sharma T, Islam N, Ahmad J, Akhtar N, Beg M (2015). Correlation between bone mineral density and oxidative stress in postmenopausal women. Indian J Endocrinol Metab.

[CR34] Sheweita SA, Khoshhal KI (2007). Calcium metabolism and oxidative stress in bone fractures: role of antioxidants. Curr Drug Metab.

[CR35] Spirlandeli AL, Deminice R, Jordao AA (2014). Plasma malondialdehyde as biomarker of lipid peroxidation: effects of acute exercise. Int J Sports Med.

[CR36] Uslu S, Uysal A, Oktem G, Yurtseven M, Tanyalcin T, Basdemir G (2007). Constructive effect of exogenous melatonin against osteoporosis after ovariectomy in rats. Anal Quant Cytol Histol.

[CR37] Wauquier F, Leotoing L, Coxam V, Guicheux J, Wittrant Y (2009). Oxidative stress in bone remodelling and disease. Trends Mol Med.

[CR38] Wei M, Yang Z, Li P, Zhang Y, Sse WC (2007). Anti-osteoporosis activity of naringin in the retinoic acid-induced osteoporosis model. Am J Chin Med.

[CR39] Witt-Enderby PA, Radio NM, Doctor JS, Davis VL (2006). Therapeutic treatments potentially mediated by melatonin receptors: potential clinical uses in the prevention of osteoporosis, cancer and as an adjuvant therapy. J Pineal Res.

[CR40] Yang F, Li J, Zhu J, Wang D, Chen S, Bai X (2015). Hydroxysafflor yellow A inhibits angiogenesis of hepatocellular carcinoma via blocking ERK/MAPK and NF-kappaB signaling pathway in H22 tumor-bearing mice. Eur J Pharmacol.

[CR41] Yang J, Wu N, Peng J, Yang X, Guo J, Yin S (2016). Prevention of retinoic acid-induced osteoporosis in mice by isoflavone-enriched soy protein. J Sci Food Agric.

[CR42] Zawilska JB, Skene DJ, Arendt J (2009). Physiology and pharmacology of melatonin in relation to biological rhythms. Pharmacol Rep.

[CR43] Zhang H, Zhao Y, Li YQ, Sun XD, Bai XY, Zhao DQ (2010). Effects of deer tendons collagen on osteoporosis rats induced by retinoic acid. Zhong Yao Cai.

[CR44] Zhang L, Su P, Xu C, Chen C, Liang A, Du K (2010). Melatonin inhibits adipogenesis and enhances osteogenesis of human mesenchymal stem cells by suppressing PPARgamma expression and enhancing Runx2 expression. J Pineal Res.

[CR45] Zhang ZM, Li ZC, Jiang LS, Jiang SD, Dai LY (2010). Micro-CT and mechanical evaluation of subchondral trabecular bone structure between postmenopausal women with osteoarthritis and osteoporosis. Osteoporosis Int.

[CR46] Zhao S, Niu F, Xu CY (2016). Diosgenin prevents bone loss on retinoic acid-induced osteoporosis in rats. Ir J Med Sci (1971-).

