# Correction to: Melatonin prevents bone destruction in mice with retinoic acid-induced osteoporosis

**DOI:** 10.1186/s10020-021-00401-4

**Published:** 2021-10-26

**Authors:** Xudong Wang, Tongzhou Liang, Yuanxin Zhu, Jincheng Qiu, Xianjian Qiu, Chengjie Lian, Bo Gao, Yan Peng, Anjing Liang, Hang Zhou, Xiaoming Yang, Zhiheng Liao, Yongyong Li, Caixia Xu, Peiqiang Su, Dongsheng Huang

**Affiliations:** 1 Department of Orthopedics, Sun Yat-Sen Memorial Hospital of Sun Yat-Sen University, #107 West Yan Jiang Road, Guangzhou, 510120 Guangdong China; 2 Department of Orthopedics, The First Affiliated Hospital of Sun Yat-Sen University, #58 Zhongshan Road II, Guangzhou, 510080 Guangdong China; 3 Research Centre for Translational Medicine, The First Affiliated Hospital of Sun Yat-Sen University, Guangzhou, 510080 Guangdong China

## Correction to: Mol Med (2019) 25:43 https://doi.org/10.1186/s10020-019-0107-0

Following publication of the original article (Wang et al. 2019), the authors identified an error in Fig. 4. The correct figure is given in this Correction article.

**Fig. 4:**
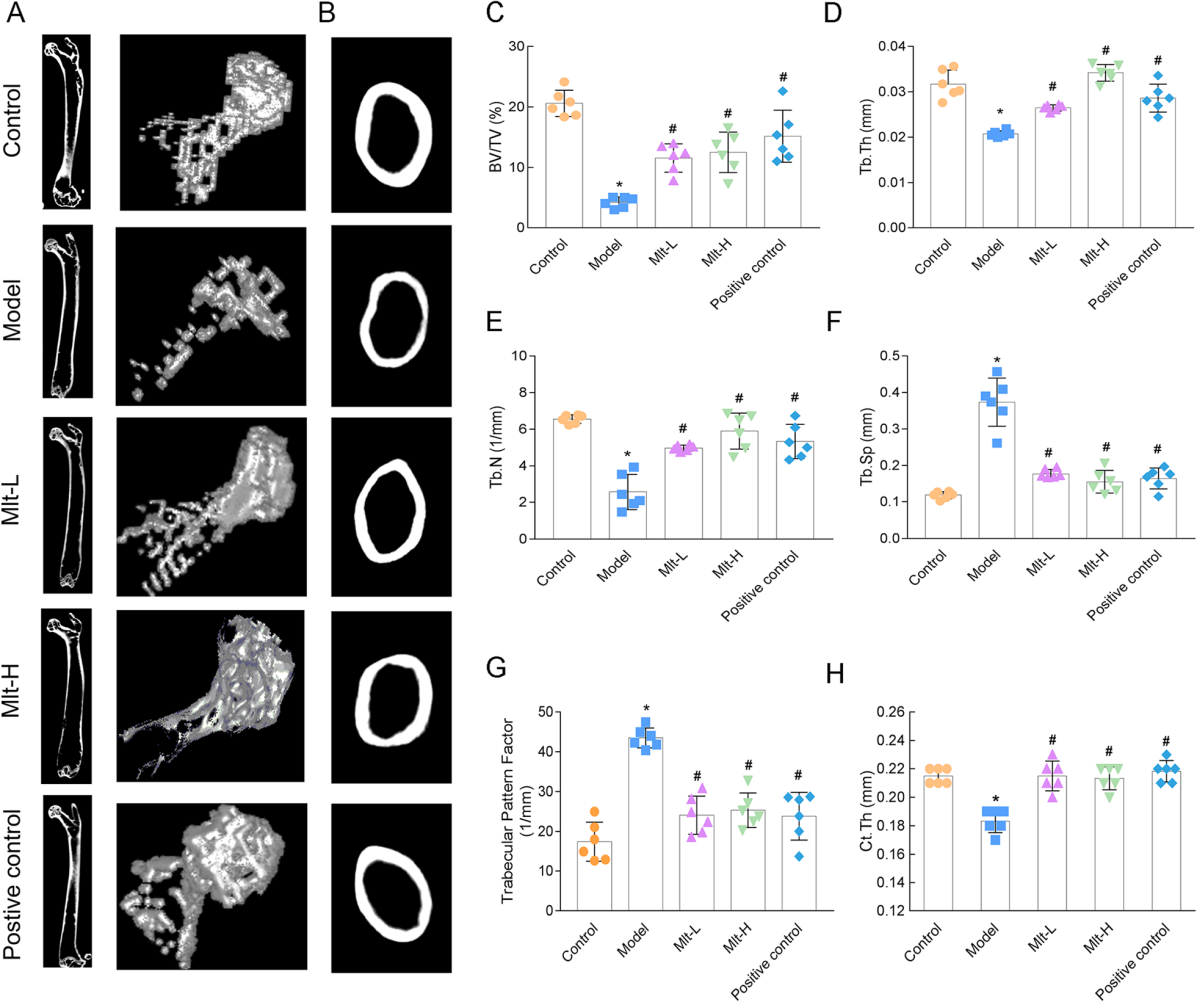
Effect of melatonin on the femur microstructure of osteoporosis (OP) model mice. **a** and **b** Micro-CT detection after the administration of melatonin or alendronate. Images of 2D and 3D reconstruction obtained by micro-CT showed that the model group had a reduction in the number of femoral trabeculae and the thickness of femur diaphysis. Microstructure parameters of BV/TV (**c**), Tb. Th (**d**), Tb. N (**e**), Tb. Sp (**f**), trabecular pattern factor (**g**), and Ct. Th (**h**) were improved in melatonin-treated mice. Control: normal mice, Model: retinoic acid (RA)-induced OP model mice, Mlt-L: low-dose melatonin-treated OP model mice, Mlt-H: high-dose melatonin-treated OP model mice. Positive control: alendronatetreated OP mice. *, P < 0.05 vs control. #, P < 0.05 vs model; n = 6 per group
