# Supplementary figures and images for: Melatonin prevents bone destruction in mice with retinoic acid–induced osteoporosis
Source: Mol Med. 2019 Aug 28;25:43. doi: 10.1186/s10020-019-0107-0 (PMC6714316; doi:10.1186/s10020-019-0107-0)

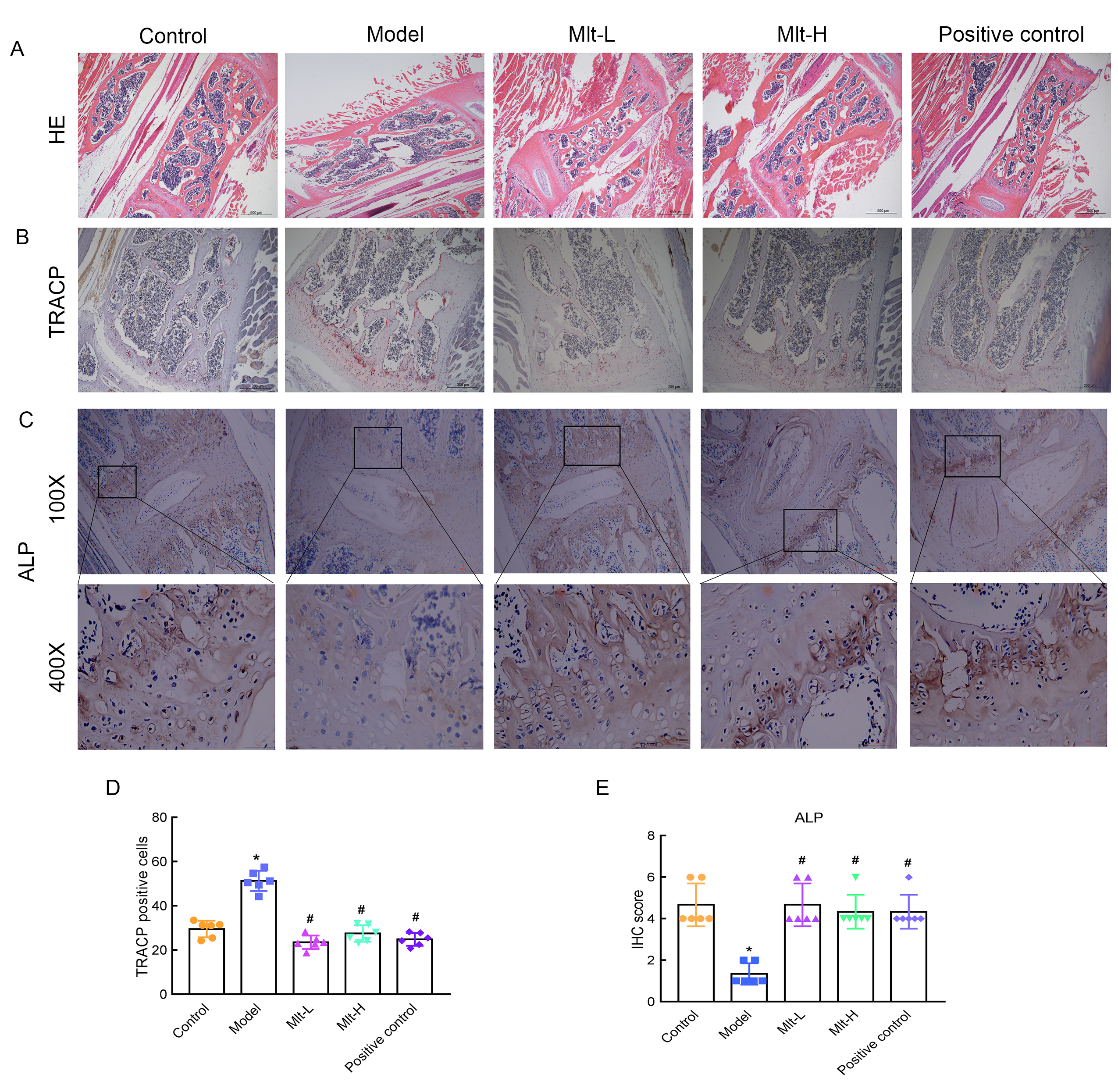

Supplement: Supplementary file 1 — Figure S1. Melatonin improves the vertebral (L1) trabecular bone and reduces the number of osteoclasts of osteoporosis (OP) model mice. After the administration of melatonin or alendronate to trabecular bone for 2 months, vertebra were examined by HE staining (40X) (A), TRACP staining (100X) (B), and ALP IHC staining (100X and 400X) (C). The number of TRACP-stained osteoclasts was quantified (D) and the IHC staining intensity was scored (E). Control: normal mice, Model: retinoic acid (RA)-induced OP model mice, Mlt-L: low-dose melatonin-treated OP model mice, Mlt-H: high-dose melatonin-treated OP model mice. Positive control: alendronate-treated OP mice. *, P < 0.05 vs control. #, P < 0.05 vs model; n = 6 per group. (TIF 8735 kb) [file 10020_2019_107_MOESM1_ESM.tif]

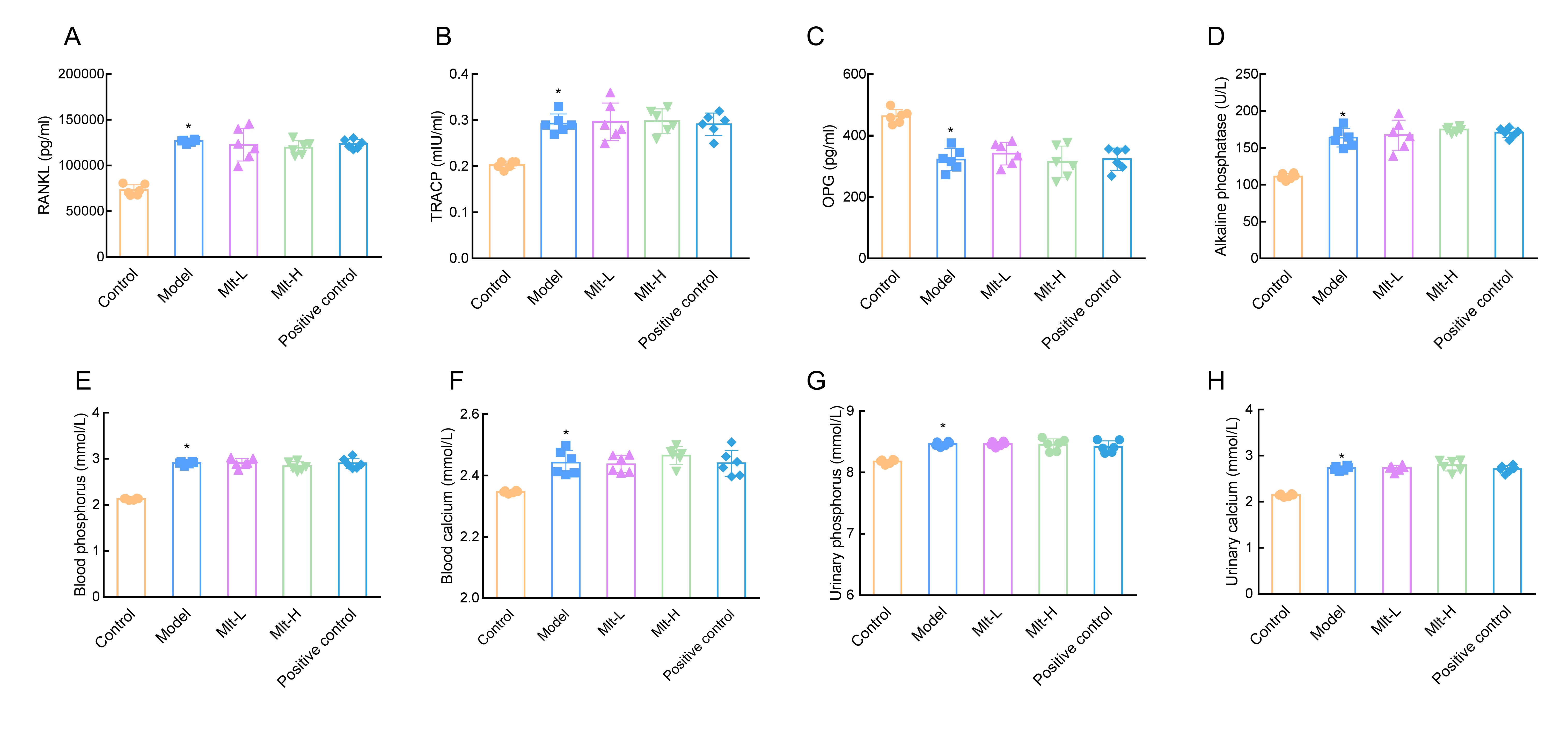

Supplement: Supplementary file 2 — Figure S2. Biochemical markers bone metabolism in osteoporosis (OP) mice. Laboratory tests for biochemical markers of bone metabolism. Serum bone metabolism-related indicators RANKL (A), TRACP (B), OPG (C), ALP (D), phosphorus (E), calcium (F), and urinary phosphorus (G), and calcium (H) were determined after the administration of retinoic acid (RA) for 15 d. Control: normal mice, Model: RA-induced OP model mice, Mlt-L: low-dose melatonin-treated OP model mice, Mlt-H: high-dose melatonin-treated OP model mice. Positive control: alendronate-treated OP mice. *, P < 0.05 vs control. #, P < 0.05 vs model; n = 6 per group. (TIF 1247 kb) [file 10020_2019_107_MOESM2_ESM.tif]
